# Comparative Analysis of Two *Helicobacter pylori* Strains using Genomics and Mass Spectrometry-Based Proteomics

**DOI:** 10.3389/fmicb.2016.01757

**Published:** 2016-11-11

**Authors:** Roger Karlsson, Kaisa Thorell, Shaghayegh Hosseini, Diarmuid Kenny, Carina Sihlbom, Åsa Sjöling, Anders Karlsson, Intawat Nookaew

**Affiliations:** ^1^Nanoxis Consulting ABGothenburg, Sweden; ^2^Department of Infectious Diseases, Sahlgrenska Academy, University of GothenburgGothenburg, Sweden; ^3^Department of Microbiology and Immunology, University of GothenburgGothenburg, Sweden; ^4^Department of Biology and Biological Engineering, Chalmers University of TechnologyGothenburg, Sweden; ^5^Proteomics Core Facility, Sahlgrenska Academy, University of GothenburgGothenburg, Sweden; ^6^Department of Biomedical Informatics, College of Medicine, University of Arkansas for Medical Sciences, Little RockAR, USA

**Keywords:** Genomics, *Helicobacter pylori*, MaxQuant, protein abundance, proteomics, TMT

## Abstract

*Helicobacter pylori*, a gastroenteric pathogen believed to have co-evolved with humans over 100,000 years, shows significant genetic variability. This motivates the study of different *H. pylori* strains and the diseases they cause in order to identify determinants for disease evolution. In this study, we used proteomics tools to compare two *H. pylori* strains. Nic25_A was isolated in Nicaragua from a patient with intestinal metaplasia, and P12 was isolated in Europe from a patient with duodenal ulcers. Differences in the abundance of surface proteins between the two strains were determined with two mass spectrometry-based methods, label-free quantification (MaxQuant) or the use of tandem mass tags (TMT). Each approach used a lipid-based protein immobilization (LPI^TM^) technique to enrich peptides of surface proteins. Using the MaxQuant software, we found 52 proteins that differed significantly in abundance between the two strains (up- or downregulated by a factor of 1.5); with TMT, we found 18 proteins that differed in abundance between the strains. Strain P12 had a higher abundance of proteins encoded by the *cag* pathogenicity island, while levels of the acid response regulator ArsR and its regulatory targets (KatA, AmiE, and proteins involved in urease production) were higher in strain Nic25_A. Our results show that differences in protein abundance between *H. pylori* strains can be detected with proteomic approaches; this could have important implications for the study of disease progression.

## Introduction

*Helicobacter pylori* is a Gram-negative, spiral shaped 𝜀-proteobacterium that colonizes the stomachs of roughly half the world’s population (Brown, 2000). Infection with *H. pylori* typically causes a mild, mixed gastritis; however, chronic infections cause severe clinical outcomes, such as duodenal and gastric ulcers, in approximately 15% of infected individuals. Gastric adenocarcinomas and mucosa-associated lymphoid tissue lymphomas occur in an additional 1–2%. Notably, the coexistence of gastric cancer and duodenal ulcer in the same individual is rare. Chronic *H. pylori* infection also contributes to gastric issues that are associated with the development of gastric cancer, such as the loss of acid secretion and the loss of acidic mammalian chitinase expression (Nookaew et al., 2013). The factors leading to this divergence in clinical outcomes are not entirely known, but host genetics that regulate the potency of the immune response toward the infection, bacterial genetics, and environmental factors, such as diet and smoking, contribute (Ahn and Lee, 2015).

The different strains of *H. pylori* are very diverse; there is a much higher frequency of recombination than point mutations. At the same time, the phylogeny of this bacterium is clearly traceable and reflects the ancestry of the carrier and the migration of ancient human groups (Falush et al., 2003; Suerbaum and Achtman, 2004; Thorell et al., 2016). During evolution, some genes remain present in all strains and make up the core genome of a species, but other genes occur variably among different isolates. It is beneficial for a pathogen to be able to adapt to new environmental niches. The genes that show the greatest sequence variations are often those that encode surface-exposed proteins, including outer membrane proteins (OMPs), that are the first to interact with the host (Suerbaum and Josenhans, 2007; Jabbour et al., 2010). The surface-exposed proteins have different functional roles; they function as adhesion factors (e.g., BabA and SabA), nutrient transporters, secreted toxins, iron-chelating proteins, and proteases, for example (Solis and Cordwell, 2011). A study comparing the frequency of recombination across the *H. pylori* genome showed that genes for OMPs are hotspots of recombination compared to, for example, metabolic genes (Yahara et al., 2012). This motivates studies that focus on how surface-exposed proteins vary in abundance across different *H. pylori* strains and how this variation affects clinical outcome.

The recently developed technology called Lipid-based Protein Immobilization (LPI^TM^) immobilizes bacteria within a flow cell, and exposed proteins are digested with an enzyme, such as trypsin (Chooneea et al., 2010). Combining LPI with mass spectrometry-based proteomics targets surface-exposed proteins for analysis. One study that performed proteomic typing of *H. pylori* strains with the LPI^TM^ technology found that 60% of the strain-specific peptides that were found to be unique biomarkers of the *H. pylori* J99 strain are membrane-associated proteins, several of which belong to the outer membrane (Karlsson et al., 2012). Different methodologies can be used to quantitatively compare samples. These methods include labeling peptides with stable isotopes in culture (stable isotope labeling with amino acids in cell culture, SILAC), labeling prior to digesting samples (isotope-coded affinity tags, ICAT), or labeling after digestion (isobaric tags for relative and absolute quantitation, iTRAQ; or tandem mass tags, TMT). TMT labeling enables the relative quantification, with good sensitivity, of uniquely labeled peptides in samples that are pooled for MS analysis (Thompson et al., 2003). MS/MS-based tag detection is often used to generate quantitative proteomic profiles between mammalian cell lines (Paulo et al., 2013). However, there are also methods and data analysis platforms, such as MaxQuant, that rely on label-free quantitation (Cox and Mann, 2008) where the amount of detected protein is derived from the sum of the peak intensities of the observed peptides. This analysis was also included in the present study.

Here, our aim was to evaluate and compare genetic content and protein abundance between two pathogenic strains of *H. pylori*; strain P12 was isolated in Europe from a patient with duodenal ulcers, and strain Nic25_A was isolated in Nicaragua from the antrum of a patient with atrophy and intestinal metaplasia. The selected whole-genome sequence of Nic25_A (Thorell et al., 2016) was compared with the complete genome sequence of *H. pylori* strain P12 (Fischer et al., 2010) to identify similarities and differences in the genomic potential. The two strains were cultured under controlled conditions and analyzed with mass spectrometry-based proteomics to compare their cell-surface proteomes.

Due to the genetic heterogeneity of *H. pylori* strains, we used the peptides found in both strains (common peptides) to enable for a better comparison of protein abundance as one part of the study. The common peptides were assayed with label-free MaxQuant analysis or with TMT labels for relative quantification. Another part of the study focused on unique proteins detected in either strain. Using these strategies, we were able to observe differences in the abundance of virulence factors between the two *H. pylori* strains that will be useful for evaluating the pathogenicity of these bacteria.

## Materials and Methods

### Data Acquisition and Analysis of Genome Sequences

The genomic information for *H. pylori* strain P12 (Fischer et al., 2010) was retrieved from the RefSeq database at NCBI. The genomic information for Nic25_A was retrieved from our previous work (Thorell et al., 2016) deposited in the Sequence Read Archive^1^) under accession number SRP04449; the sequence of Nic25_A, from a patient with intestinal metaplasia and atrophy, (SRS679647) was used in this project.

### Comparative Genomics

To ensure that the annotation between both genomes was compatible for comparative analyses, we used the Prokka pipeline v1.9 (Seemann, 2014) to annotate both genomes. This pipeline predicts open reading frames (ORFs) using Prodigal (Hyatt et al., 2010), specifically developed for Gram-negative bacteria; it also allows for rRNA, tRNA, and signal-peptide prediction. Prokka was applied to the assembled contigs of the Nic25_A strain and the P12 chromosome and plasmid. As the primary annotation source in Prokka, we used the strain 26695 genome with the most recent reannotation (Resende et al., 2013) and with manually curated OMP annotation (Alm et al., 2000). For predicted ORFs with no closely related (blastp *e*-value < 10^-9^) match in the 26695 genome, annotation was based on the global *H. pylori* reference databases (see **Supporting Files 6** for complete annotation of the two strains). To compare the genome sequences of the two strains, we used MAUVE (Darling et al., 2010) to perform entire-genome pairwise alignment, and the alignment was visualized with BRIG (Alikhan et al., 2011). The protein-coding sequences of the two genomes were further clustered to identify groups of orthologous proteins with the UClust software (identity threshold of 0.8) (Edgar, 2010). Then, the representative protein sequences of each cluster were further predicted for their membrane-associated properties with LipoP v1.0 (Juncker et al., 2003) and PSORTb v3.02 (Yu et al., 2010).

### *Helicobacter pylori* Strain Isolation and Cultivation

*Helicobacter pylori* strains P12 and the Nicaraguan clinical isolate Nic25_A were cultured on Columbia blood agar plates under microaerophilic conditions (80% N_2_, 10% CO_2_, 10% H_2_), which is standard for *H. pylori* (Janzon et al., 2009). The strains were replated and cultured for 24 h before the colony biomass was scraped from the plates and suspended in 500 μL of phosphate buffered saline.

### Proteomics – Sample Processing and Generation of Peptides with LPI^TM^ HexaLane

**FIGURE 1:**
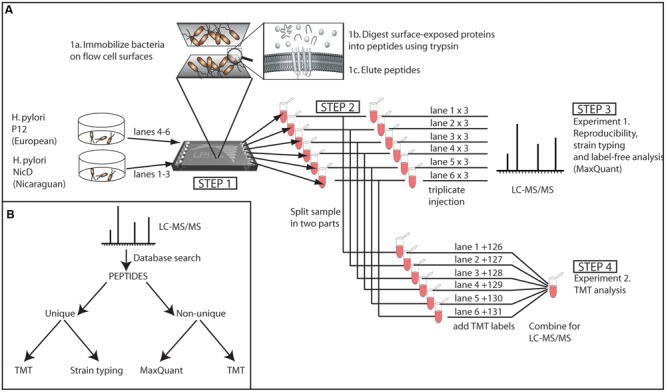
**(A)** Experimental setup. The two strains were grown under the same conditions, washed, and each injected into three lanes of the LPI^TM^ flow cell (Step 1). The bacteria were allowed to become immobilized on the flow cell surface (1a). The immobilized bacteria were treated with trypsin to generate peptides from the exposed proteins (1b), and the peptides were eluted from the flow cell channels (1c). The trypsin-digested protein sample from each lane was split into two parts (Step 2) and analyzed in triplicate LC-MS/MS injections (Step 3) or labeled separately for combined TMT semiquantitative LC-MS/MS (Step 4). **(B)** Data analysis strategy.

An overview of the workflow for sample processing and peptide generation is presented in **Figure 1**. The bacterial biomass for each strain was washed with PBS, centrifuged for 8 min at 4000*g*, and resuspended in PBS. This procedure was repeated three times before the bacteria were resuspended in PBS to an optical density (600 nm) of approximately 0.5 (NanoDrop-1000 Spectrophotometer, Thermo Fisher Scientific). The bacterial suspension was immediately loaded into the LPI^TM^ HexaLane FlowCell (Nanoxis Consulting AB) (**Figure 1**, step 1). An excess of bacteria was applied to the flow cell by adding a bacterial suspension (100 μl) to fill the LPI^TM^ Flow Cell channel, which has a volume of 50 μl. Excess bacterial suspension was removed from the inlet and outlet ports to ensure that there was an equal amount of bacterial material in each flow cell channel. The flow cells containing bacteria were incubated for 2 h at room temperature to allow cells to attach. The channels were subsequently washed with 1.0 mL of triethylammonium bicarbonate (TEAB) buffer (100 mM) to remove excess, unbound bacteria. Bacterial membrane proteins were digested by injecting 100 μL of trypsin (20 μg/mL) into the LPI^TM^ HexaLane FlowCell channels and incubating for 30 min at room temperature.

The cleaved peptides were eluted by injecting TEAB buffer (200 μl, 100 mM) into the channels and collecting the flow-through at the outlet ports with a pipette. The eluate was incubated overnight at 37°C to allow for complete digestion. Afterward, the samples were frozen at -20°C until analysis by MS. The peptide samples were split into two equal parts and analyzed by two different approaches. One aliquot was analyzed by LC-MS/MS, as described previously (Karlsson et al., 2012), in order to generate protein lists for each sample; each sample was analyzed in triplicate injections (**Figure 1**, step 3). Relative quantification of peptides was performed on the second aliquot with Tandem Mass Tag (TMT), MS-based quantification (**Figure 1**, step 4).

### Mass Spectrometry-Based Analysis of Peptides

The peptide samples were analyzed with two different LC-MS/MS approaches using the LTQ-Orbitrap XL at the Proteomics Core Facility, University of Gothenburg^2^), as described previously (Karlsson et al., 2012) (with some modifications). The length of the MS analysis was 120 min, and the LC gradient was adjusted to increase from 5 to 37% acetonitrile during 100 min. The samples were first analyzed individually in triplicate injections, (**Figure 1**, step 3). In addition to this, we quantified the relative abundance of peptides with Tandem Mass Tag (TMT), MS-based quantification using NHS chemistry to target primary amines (Proteome Science, Surrey, UK) (**Figure 1**, step 4). For quantifying the relative abundance of proteins, the LTQ-Orbitrap XL was switched between CID (collision-induced dissociation) and HCD (high-energy collision dissociation) in the data-dependent mode of the three most abundant doubly, triply, and quadruply protonated ions from each FT-MS scan. The CID scan was used for identification and the HCD scan was used to quantify the TMT reporter ions. The settings for the MS2 was as follows: 1 microscan for HCD-MS2 at 7500 resolution (at m/z 400), mass range m/z 100–2000 with a collision energy of 50%, 1 microscan for CID-MS2 with a collision energy of 30%. Dynamic exclusion of a precursor selected for MS2 was used for 60 s after one repeat. The TMT-labeled sample was analyzed two additional times with an exclusion list of all m/z already passing the peptide identification criteria in order to increase the number of identified proteins. All raw files generated in this study can be found in the PRIDE database under accession number PXD005074.

### Protein Quantification with TMT

MS/MS data were analysed with Proteome Discoverer version 1.4 (Thermo Fisher Scientific) incorporating Mascot version 2.3 (Matrix Science, London, UK). Mascot was configured to search against the custom-made database of the Nic25_A and P12 proteomes (3,066 total entries). The MS peptide tolerance was set as 10 ppm, MS/MS tolerance as 0.5 Da, trypsin digestion allowing 1 missed cleavage with variable modifications, methionine oxidation, and, where relevant, fixed modifications N-terminal TMT6-plex label, lysine TMT6-plex label. The detected peptide threshold in the software was set to a detection confidence using a false discovery rate (FDR) of 1% (the FDR determined via target-decoy). For quantification, the ratios of TMT reporter ion intensities in MS/MS spectra (m/z 126.12, 127.13, 128.13, 129.14, 130.14) from raw data sets were used to calculate fold changes for common peptides (not unique) that are isoforms or proteins of the same family between the strains. Peptide measurements were not grouped, missing values were replaced, and normalization was used. The ratios were then exported into Microsoft Excel for manual data interpretation.

### Label-Free Protein Quantification

Individual raw sample files were processed with MaxQuant (version 1.5.0.30). The data from the three samples of each strain were searched against a custom database compiled from the Prokka-annotated protein FASTA files for each respective strain (i.e., one database for P12 and one for Nic25_A). The searches were split into separate experiments, with each experiment consisting of the raw files corresponding to three injections per sample. The variable modifications for the searches were acetyl (N-terminal) and oxidation (methionine), with a maximum of five modifications per peptide. The digestion enzyme was trypsin, and a maximum of one missed cleavage was accepted. For the primary search, the precursor peptide tolerance was 10 ppm, and the MS/MS tolerance was 0.5 Da. The match between runs was selected for each experiment with a match time window of 1 min. A minimum of razor peptide was required for identification, and a minimum score of 40 was accepted for modified peptides. The FDR was set to 0.01 for peptide spectral matches, proteins and site and calculated using reverse decoy. The maximal peptide PEP was 0.01. Minimal peptide length was seven amino acids.

Protein quantification was performed using intensity-based absolute quantification (iBAQ), which sums the peak intensities of all matching peptides and divides this by the number of theoretical observable peptides to provide a proxy quantification of the protein levels (Schwanhausser et al., 2011). The minimum reporter PIF (precursor intensity fraction) was 0.75. The percentage of each protein in the pool was determined by dividing the individual iBAQ value for each protein by the summed iBAQ values of all the quantified proteins identified in the sample. The three individual P12 and Nic25_A samples were grouped, and the coefficient of variation and median percentage were determined.

### *H. pylori* Strain Identification with Peptide Mapping

One hundred and sixty genome sequences of *H. pylori* strains (complete and draft) were retrieved from RefSeq and the amino acid sequences were collected and combined with the amino acid sequences of *H. pylori* Nic25_A to become a database for peptide mapping. The high quality peptides identified with mass spectrometry were mapped using blast+ v2.2.27 (blastp, word size = 2, score matrix = PAM30). The results from blast+ were further used for strain-typing analysis with the scoring method proposed by Karlsson et al. (2012).

### Protein Abundance Analysis

Protein intensities from MaxQuant for P12 and Nic25_A were combined into the same file for differential-abundance analysis with the 26695 gene IDs. If the protein was annotated with the same gene ID in the P12 and Nic25_A, respectively, the intensities were tested for differential abundance against each other with the Qprot algorithm (Choi et al., 2008). This was also used for identifying proteins uniquely abundant in each strain. The criterion set for this analysis was: protein found in at least 2 of 3 runs (triplicate analysis) for one strain but absent in all three runs in the other strain.

## Results and Discussion

### *H. pylori* Nic25_A Genome Properties and Its Comparison with *H. pylori* P12

**FIGURE 2:**
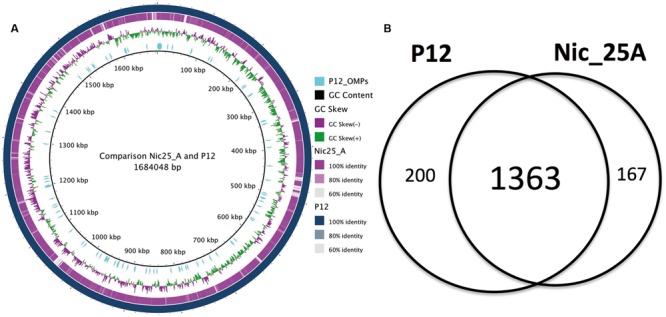
**Comparative genome analysis between Nic_25A and P12. (A)** Comparison between the Nic_25A and P12 genomes illustrated with a BRIG plot. The outermost ring represents the P12 chromosome, and the second, purple ring represents the alignment of the Nic25_A draft genome contigs. The third ring from the outside shows the GC-skew between the genomes. The cyan lines mark the distribution of outer membrane protein (OMP) genes (as defined by Alm et al., 2000 and Voss et al., 2014). **(B)** A Venn diagram compares the core and pan-genomes of Nic_25A and P12 based on orthologous clustering with 0.8 identity cut-off.

Based on the genomic information, the Nic25_A genome was found to contain 1627 predicted ORFs, similar to other *H. pylori* strains. The *H. pylori* Nic25_A genome was further compared to the *H. pylori* P12 genome through entire genome alignment with MAUVE (Darling et al., 2010) to produce a BRIG plot (**Figure 2A**). Several single nucleotide variants (SNVs) between the two *H. pylori* strains were distributed along the chromosome and accounted for about 1.1% of the total nucleotides in the *H. pylori* P12 chromosome (**Supporting Files 1**–**3**).

The amino acid sequences of the two strains were subjected to orthologous clustering with UClust (Edgar, 2010) to identify protein families (**Figure 2B**). Using an identity cut-off of 0.8, we identified 1730 pan-protein families with 200 (P12) or 167 (Nic25_A) unique protein sequences (**Supporting Files 1**–**3**). Of these, 1363 proteins were found in common between the two strains. In total, 53 proteins were identical between the two strains; among these were OMPs, HorE, HopK, HopN, SabB, and LPP20.

### Unique Peptides Predicted from Bioinformatics Analyses are Identified with MS

**FIGURE 3:**
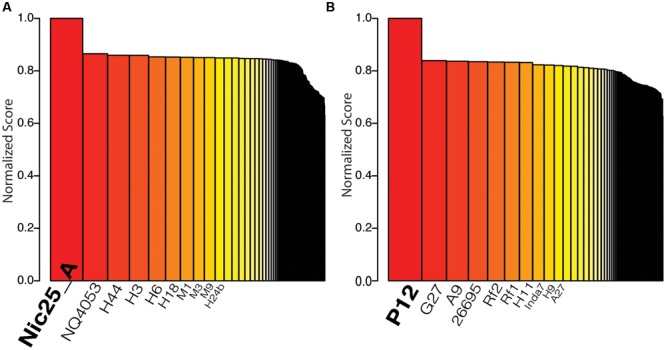
**Strain typing results using the method proposed by Karlsson et al., 2012.** Bar plot of sorted normalized score based on the collection of 160 genomes from different *Helicobacter pylori* strains. The strains with the top ten highest scores are labeled on the *x*-axis. **(A)** The result when peptide data from *H. pylori* Nic25_A was used for calculating the score. **(B)** The result when peptide data from *H. pylori* P12 was used for calculating the score.

More than half of the membrane-associated protein families identified by bioinformatics analysis were also identified with high-quality peptides using an identity cut-off of 0.99. The high-quality peptides derived from individual LPI^TM^ lanes and individual MS injections were used to detect unique peptides from the two strains. Proteomic strain-typing based on unique peptides was performed according to Karlsson et al. (2012) (i.e., by matching the identified peptides to the genomes of different *H. pylori* strains and ranking the results according to the highest number of matched peptides per strain). Thus, the analysis of the proteomic results verifies that the top-ranked strain is the strain being analyzed, as illustrated for the strain ranking analysis for Nic25_A and P12 (**Figure 3**). We achieved high reproducibility when comparing different individual injections or different LPI^TM^ lanes with a minimum Spearman rank correlation of 0.83 (**Supplementary Figure S1**).

One factor contributing to the demonstrated reproducibility is that the same amount of material is immobilized in each lane, due to the same capturing surface-area of each lane. This demonstrates one of the benefits of using the LPI approach for comparative proteomics.

### Analysis of Differential Protein Abundance

**FIGURE 4:**
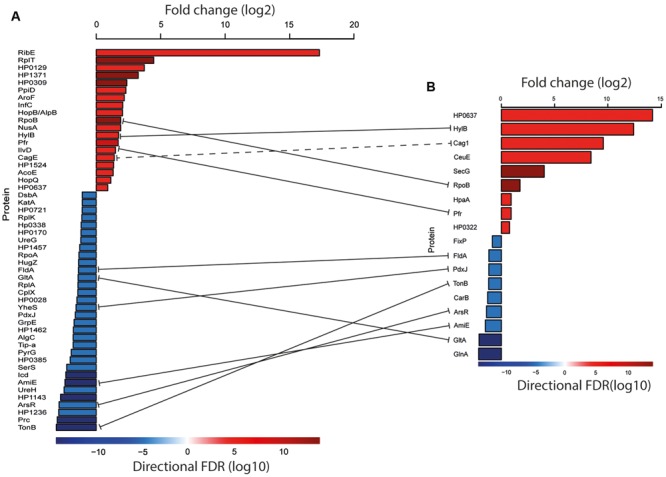
**Proteins that differed significantly in abundance between the two strains (FDR 0.05). (A)** Proteins identified with the MaxQuant label-free approach. **(B)** Proteins identified with the TMT approach. Red bars indicate proteins more abundant in P12; blue bars indicate proteins more abundant in Nic25_A. The values for the fold change are given as log_2_ (upper bar). Lower bar colors represent log_10_ FDRs and are reflected in the bar graphs. The solid lines represent the common proteins that were found in label-free and TMT approaches. A dashed line represents proteins that come from the same *cag* operon. Processed data can be found in **Supporting Files 4** and 5.

Using the protein intensities calculated from the MaxQuant and TMT analyses as input into the QProt software, we identified 52 proteins that differed in abundance (FDR 0.05) between Nic25_A and P12 using the MaxQuant data and 18 proteins using the TMT data (**Figure 4**). Processed data can be found in **Supporting Files 4** and **5**.

### Analysis of Unique Proteins

In order to detect proteins that were uniquely abundant in either of the two strains, we used MaxQuant to evaluate the triplicate runs with the following criteria: unique for Nic25_A—found in 2 out of the 3 runs for Nic25_A but in 0 of the P12 runs; unique for P12—found in 2 out of the 3 runs for P12 but in 0 of the Nic25_A runs. This produced 59 unique proteins for both Nic25_A and P12 (**Supporting Files 7** and **8**).

### Workflow of Comparative Proteomics of the Two *H. pylori* Strains

The goal of this study was to evaluate the feasibility of using MS-based proteomics (and genomic analyses) to quantify differences in protein abundance in the human pathogen *H. pylori*, which displays high intraspecies sequence variability. The driving hypothesis is that it is possible to identify differences in protein abundance between *H. pylori* strains isolated from different clinical and geographical origins, and this information can be used to evaluate biologically important features. To demonstrate proof-of-principle, we chose two *H. pylori* strains isolated from patients with different clinical manifestations and from different geographical locations. The P12 strain has been analyzed in several studies and is sequenced. We isolated and sequenced strain Nic25_A previously (Thorell et al., 2016). We used these genome sequences to determine unique peptide/protein markers for each specific strain, and we quantified the relative abundance of proteins in both strains.

Using the LPI^TM^ HexaLane FlowCell and the TMT 6-plex for relative quantification proved to work well, as did the label-free approach. The LPI HexaLane FlowCell has six channels for immobilizing bacteria, and all six samples can be processed and analyzed in parallel. All of the channels have the same surface area for capturing bacteria; therefore, the channels bind the same amount of sample in each channel, enabling us to reliably compare proteins levels. In this study, three channels were used for *H. pylori* Nic25_A and three for *H. pylori* P12. We found that the data generated from the three replicates were highly reproducible, hence it was possible to produce statistically significant data on protein abundance using the flow cells.

A factor clearly complicating the analysis of data from shotgun proteomics approaches is the problem of annotation. Alm et al. (2000) did the first thorough annotation of *H. pylori* OMPs in 2000; they compared strains J99 and 26695, the only strains for which complete sequences were available (Alm et al., 2000). Despite this effort, the current databases are filled with divergent annotations, and it requires significant effort to identify homologies and corresponding locus names between strains. The fact that comparative proteomics and genomics rely on well-annotated databases is a major hurdle. To overcome this, we used the recently reannotated genome of strain 26695 (Resende et al., 2013) as a reference for our annotation of both the P12 and Nic25_A proteomes with the Prokka annotation pipeline (Seemann, 2014). Because we are focusing on membrane-bound and surface exposed proteins, we also curated the reference manually with regards to the OMPs described by Alm et al. (2000). Using the two newly annotated proteomes, we searched the peptides from Nic25_A injections against the Nic25_A proteome and the P12 peptides against the P12 proteome. This allowed us to compare the intensities of the detected peptides with the harmonized annotation and not introduce bias into the analysis (due to ambiguities in matching to the database).

### The Biological Significance

We found that the abundance of several known virulence factors and OMPs differed between strains P12 and Nic25_A. Based on the shared protein hits and relative quantification, *cag* pathogenicity island (CagPAI) proteins were more abundant in P12 than Nic25_A as shown in **Figure 4**. CagA is a well-described *H. pylori* virulence factor that is injected into mammalian cells through a type IV secretion system encoded by several other ORFs in the CagPAI. CagE is an essential component of this secretion system and, based on homology, functions as an ATPase. Loss of CagE leads to the incomplete assembly of the secretion system (Zhang et al., 2002). As seen in the **Figure 4**, the adhesion factors AlpB, HpaA, and HopQ were also found to be more abundant in the P12 strain. Based on the unique proteins identified in the triplicate runs (**Supporting File 8**), the adhesins BabB and SabB were more abundant in the P12 strain, as were OMPs of the Hop and Hom families (Voss et al., 2014) and other virulence factors including vacuolating cytotoxic protein A (VacA), elongation factor (EF-Tu), and flagellar basal body protein FliL.

With respect to Nic25_A, the transcription factor ArsR was more abundant in this strain than in P12 as shown in **Figure 4**; ArsR is part of a two-component acid-response system (Wen et al., 2003). The Nic25_A isolate was also more abundant in several other major virulence factors; these include UreG and UreH, two urease accessory proteins, and KatA, involved in the protection against reactive oxidative species that is also regulated by ArsR (Wen et al., 2003). From the list of unique proteins found in Nic_25A, there were also some known virulence factors present (**Supporting File 7**), including UreB and heat shock protein GroEL. It is interesting to note that the Nic25_A strain, isolated from an individual with atrophy and intestinal metaplasia and commonly thought to be associated with hypochlorhydria (pH 4–7), overexpressed several genes involved in acid resistance and oxidative stress compared to P12, which was isolated from a patient with duodenal ulcer. Our results are, however, corroborated by other proteome studies where gastric cancer is associated with a higher abundance of urease genes and antioxidant proteins such as KatA and TrxA, as well as GroEL (Park et al., 2006; Momynaliev et al., 2010; Repetto et al., 2014). Thus, the increase in ArsR levels might be associated with a greater capacity to respond to oxidative stress rather than pH changes.

## Conclusion

In this study, we analyzed two strains as a proof-of-concept that, with proper controls for annotation and the identification of peptide hits, an LC-MS-based proteomics approach is useful for detecting differences in *H. pylori* proteomes. Importantly, we want to stress that the high degree of strain variability among *H. pylori* protein sequences requires each strain to be compared to its own sequences with correct annotations for proper detection and accurate quantification of surface proteins.

## Author Contributions

All authors (RK, KT, SH, DK, CS, ÅS, AK, and IN) contributed to the writing of this manuscript, and all authors have given approval to the final version of this manuscript.

## Conflict of Interest Statement

Roger Karlsson and Anders Karlsson are part-time employees at Nanoxis Consulting AB that holds the patent rights for the LPI technology described in this manuscript. All the other authors declare that the research was conducted in the absence of any commercial or financial relationships that could be construed as a potential conflict of interest.

## Footnotes

1 http://www.ncbi.nlm.nih.gov/sra

2 http://www.proteomics.cf.gu.se

## Supplementary Material

FIGURE S1 **High reproducibility was observed when comparing different individual injections and LPI lanes with a minimum Spearman rank correlation between 0.83 and 0.94.** (Top left) Three different LPI sample lanes (Lanes 1, 2, and 3) for *H. pylori* D (corresponding to Nic25_A) and the corresponding three injections (D1.1, D1.2, D1.3; D2.1, D2.2, D2.3; D3.1, D3.2, and D3.3) for each LPI sample lane. (Top right) Corresponding LPI sample lanes (Lanes 1, 2, and 3) for *H. pylori* P12 and the corresponding three injections (P1.1, P1.2, P1.3; P2.1, P2.2, P2.3; P3.1, P3.2, and P3.3) for each LPI sample lane.

Supporting File 1 **Core genome of Nic25_A and P12**.

Supporting File 2 **Unique genes of Nic25_A**.

Supporting File 3 **Unique genes of P12**.

Supporting File 4 **Processed data MaxQuant**.

Supporting File 5 **Processed data TMT**.

Supporting File 6 **Prokka translation**.

Supporting File 7 **Unique proteins found in Nic25_A**.

Supporting File 8 **Unique proteins found in P12**.
