# Supplementary figures and images for: Comparative Analysis of Two Helicobacter pylori Strains using Genomics and Mass Spectrometry-Based Proteomics
Source: Front Microbiol. 2016 Nov 11;7:1757. doi: 10.3389/fmicb.2016.01757 (PMC5104757; doi:10.3389/fmicb.2016.01757)

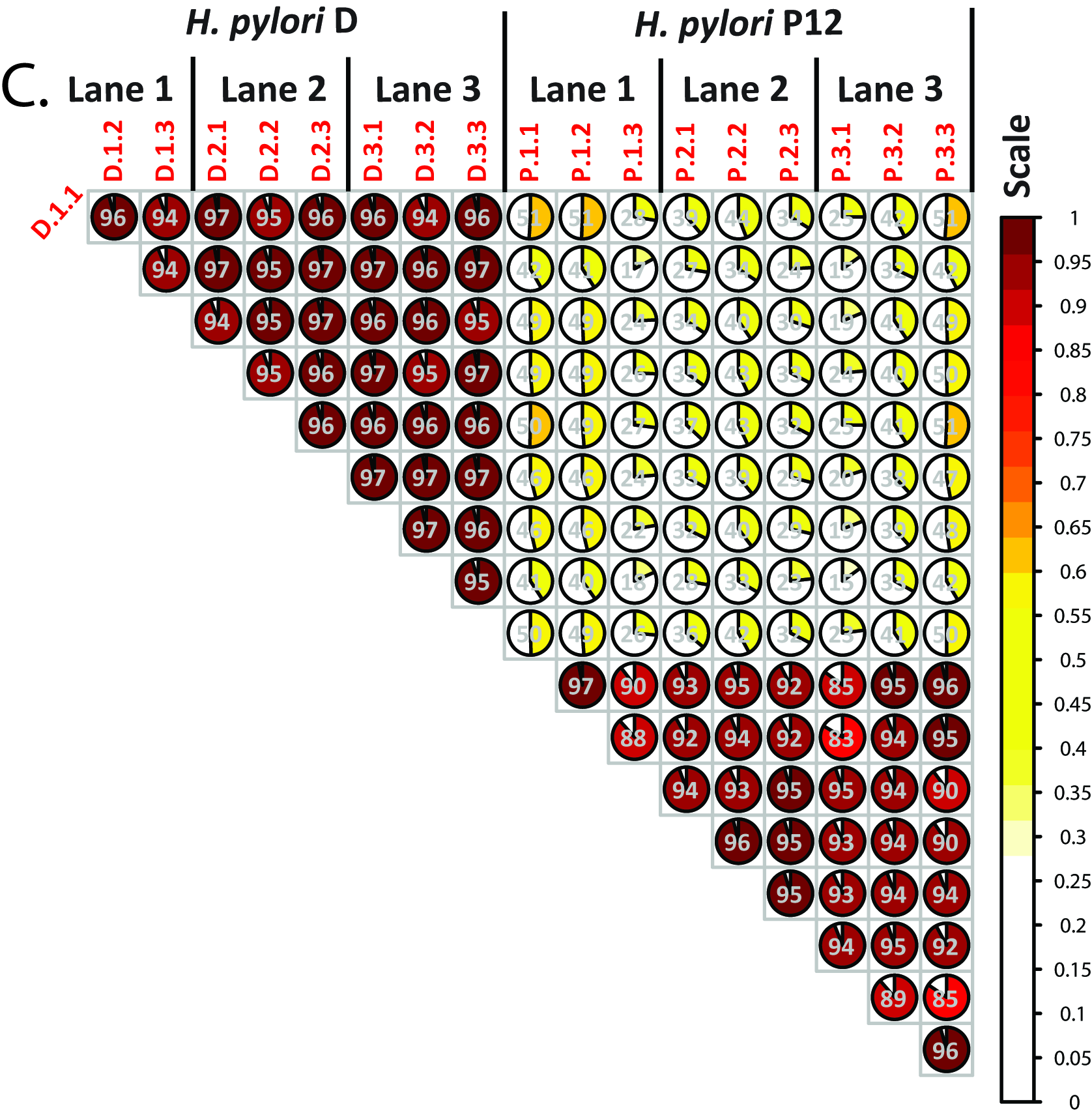

Supplement: FIGURE S1 — High reproducibility was observed when comparing different individual injections and LPI lanes with a minimum Spearman rank correlation between 0.83 and 0.94. (Top left) Three different LPI sample lanes (Lanes 1, 2, and 3) for H. pylori D (corresponding to Nic25_A) and the corresponding three injections (D1.1, D1.2, D1.3; D2.1, D2.2, D2.3; D3.1, D3.2, and D3.3) for each LPI sample lane. (Top right) Corresponding LPI sample lanes (Lanes 1, 2, and 3) for H. pylori P12 and the corresponding three injections (P1.1, P1.2, P1.3; P2.1, P2.2, P2.3; P3.1, P3.2, and P3.3) for each LPI sample lane. [file Data_Sheet_1.ZIP › Supplementary/Supplementary Figure 1_.tif]
